# Group 11 Metal Compounds with Tripodal Bis(imidazole) Thioether Ligands. Applications as Catalysts in the Oxidation of Alkenes and as Antimicrobial Agents

**DOI:** 10.3390/molecules16086701

**Published:** 2011-08-08

**Authors:** Fangwei Liu, Reema Anis, Eunmi Hwang, Rafael Ovalle, Armando Varela-Ramírez, Renato J. Aguilera, María Contel

**Affiliations:** 1 Department of Chemistry, Brooklyn College and The Graduate Center, The City University of New York, Brooklyn, NY 11210, USA; 2 Department of Biology, Brooklyn College, The City University of New York, Brooklyn, NY 11210, USA; 3 Department of Biological Sciences, The University of Texas at El Paso, El Paso, TX 79968, USA

**Keywords:** tripodal bis(imidazol) thioether pincer ligands, group 11 metals, oxidation alkenes, antimicrobial, non-toxic

## Abstract

New group 11 metal complexes have been prepared using the previously described tripodal bis(imidazole) thioether ligand (*N*-methyl-4,5-diphenyl-2-imidazolyl)_2_C(OMe)C(CH_3_)_2_S(*tert*-Bu) ({BIT^OMe,StBu^}, **2**). The pincer ligand offers a N_2_S donor atom set that can be used to coordinate the group 11 metals in different oxidation states [Au^I^, Au^III^, Ag^I^, Cu^I^ and Cu^II^]. Thus the new compounds [Au{BIT^OMe,StBu^}Cl][AuCl_4_]_2_ (**3**), [Au{BIT^OMe,StBu^}Cl] (**4**), [Ag{BIT^OMe,StBu^}X] (X = OSO_2_CF_3_^-^ **5**, PF_6_^-^ **6**) and [Cu{BIT^OMe,StBu^}Cl_2_] (**7**) have been synthesized from reaction of **2** with the appropriate metal precursors, and characterized in solution. While attempting characterization in the solid state of **3**, single crystals of the neutral dinuclear mixed Au^III^-Au^I^ species [Au_2_{BIT^OMe,S^}Cl_3_] (**8**) were obtained and its crystal structure was determined by X-ray diffraction studies. The structure shows a Au^III^ center coordinated to the pincer ligand through one N and the S atom. The soft Au^I^ center coordinates to the ligand through the same S atom that has lost the *tert*-butyl group, thus becoming a thiolate ligand. The short distance between the Au^I^-Au^III^ atoms (3.383 Å) may indicate a weak metal-metal interaction. Complexes **2-7** and the previously described Cu^I^ compound [Cu{BIT^OMe,StBu^}]PF_6_ (**9**) have been evaluated in the oxidation of biphenyl ethylene with *tert-*butyl hydrogen peroxide (TBHP) as the oxidant. Results have shown that the Au^I^ and Ag^I^ complexes **4** and **6** (at 10 mol % loading) are the more active catalysts in this oxidative cleavage. The antimicrobial activity of compounds **2**-**5**, **7** and **9** against Gram-positive and Gram-negative bacteria and yeast has also been evaluated. The new gold and silver compounds display moderate to high antibacterial activity, while the copper derivatives are mostly inactive. The gold and silver complexes were also potent against fungi. Their cytotoxic properties have been analyzed *in vitro* utilizing HeLa human cervical carcinoma cells. The compounds displayed a very low cytotoxicity on this cell line (5 to 10 times lower than cisplatin) and on normal primary cells derived from C57B6 mouse muscle explants, which may make them promising candidates as potential antimicrobial agents and safer catalysts due to low toxicity in human and other mammalian tissues.

## 1. Introduction

In the last few years the use of mixed-donor pincer ligands has increased due to the catalytic potential of their metal complexes in different organic transformations [1]. They are important in homogenous catalysis since they provide stabilization and temporary coordinative saturation to the metal center via chelation until an active site is required. A large number of donor ligands with many donor type permutations (C, O, S, N, P, *etc*…), charge and coordination number have been described [2]. Among these various combinations, mixed-donor ligands of the type N,S are particularly interesting. They are currently used as modeling active sites for metalloenzymes [3] and they have found applications in homogeneous catalysis [4] and medicinal chemistry (brain-imaging [5], antimicrobial properties [6]). They display a hard donor (N) and a soft donor (S) atom that can coordinate to a wide range of metals. They can bind to the same metal in a particular coordination state providing a different bond strength and lability for each donor atom. There is also a current interest in the incorporation of these N,S donor atoms (usually the ligands contain combinations of thiolato or thioether functional groups and amines, pyrazolyl or imidazole groups) in macrocycles [7] and in polydentate and tripodal ligands [3,4,5,6,8] in order to probe the biologically relevant coordination chemistry, the supramolecular chemistry (and metal ion recognition), or the catalytic reactions of different metals.

Recently, biomimetic tripodal bis(imidazole)thioether (BIT) ligands have been reported by Nicholas *et al*. incorporating biologically most relevant N-imidazole groups which display a significantly different basicity and donor-acceptor properties with respect to the other common nitrogen ligands (such as amines and pyrazolyl ligands) [9,10,11]. These ligands provide a sterically encumbered environment that could prevent, in principle, the formation of bimetallic species. 

**Scheme 1:**
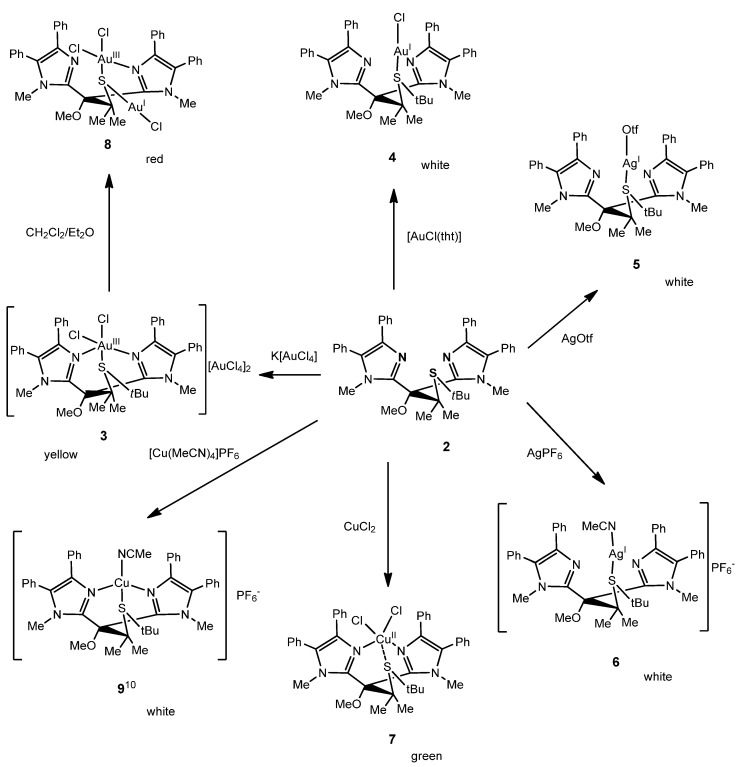
Preparation of new gold, silver and copper complexes (**3**-**7**) and previously described copper(I) **9** [10] incorporating the pincer BIT^OMe,StBu^ ligand **2** [10].

In the search for copper complexes that accurately mimic the electronic and features of the Cu_M_ site of copper hydroxylase enzymes, compounds of Cu^I^ have been successfully synthesized with these tripodal BIT ligands (Scheme 1) [9,10,11]. Their reactivity towards dioxygen was studied and new dimeric hydroxo-copper(II) derivatives were obtained. In order to incorporate the ligand set of the CuM site of the copper hydroxylase enzymes, the BIT ligands were modified according to the imidazole C(Ph or H) and N(H or Me) substituents as well as the positions (2- or 4-) of the tripodal attachment [11]. The authors concluded that N-Me substitution and 4-tethering on the imidazole unit increased oxidation and oxygenation reactivity on the derived Cu^I^ complexes while Ph-substitution and 2-tethering decreased reactivity [11].

We thought that some of these tripodal ligands (specifically BIT^OR,SR’^) could be useful in the synthesis of group 11 metal complexes with the metals in different oxidation states. We wanted a chelating (pincer) system able to stabilize group 11 metal derivatives, but with basicity and electronic properties different from those of amines. We were especially interested in the preparation of gold complexes in different oxidation states (I and III) with the same set of ligands (or same set of mixed-donor atoms within the ligand) to study their catalytic (oxidation reactions) and biological (cytotoxic, antimicrobial) behavior as a function of the oxidation state of the gold center. We also wanted to study the activity of different metals from the same triad with the same ligand. We report here on the preparation and characterization of group 11 metal complexes with previously described potentially pincer tripodal bis(imidazole) thioether ligand (N-methyl-4,5-diphenyl-2-imidazolyl)_2_C(OMe)C(CH_3_)_2_S(*tert*-Bu) {BIT^OMe,StBu^} **2**. Thus, new compounds [Au{BIT^OMe,StBu^}Cl][AuCl_4_]_2_ (**3**), [Au{BIT^OMe,StBu^}Cl] (**4**), [Ag{BIT^OMe,StBu^}X] (X = OSO_2_CF_3_^-^ **5**, PF_6_^-^ **6**) and [Cu{BIT^OMe,StBu^}Cl_2_] (**7**) have been synthesized from reaction of **2** (Scheme 1) with the appropriate metal precursors, and characterized in solution. Their catalytic activity in the oxidation of biphenyl ethylene with *tert-*butyl hydrogen peroxide (TBHP) as the oxidant has been studied. Au^I^ and especially Ag^I^ complexes displayed moderate catalytic activity. The antimicrobial activity of compounds **2**-**5** and **7** and ligand **2 ** [10] and [Cu{BIT^OMe,StBu^}(NCCH_3_)]PF_6_ (**9**) [10] were tested against Gram-positive and Gram-negative bacteria and yeast. The cytotoxic properties of these novel compounds were analyzed *in vitro* utilizing HeLa human cervical carcinoma cells. Au^I^, Au^III^ and Ag^I^ derivatives displayed high to moderate antibacterial/antifungal activity, while all the compounds were less cytotoxic against the HeLa tumor cells than cisplatin and even less toxic to normal primary cells derived from C57B6 mouse muscle explants.

## 2. Results and Discussion

### 2.1. Chemistry

BIT ligands with bulkier S-alkyl groups (*tert*-Bu or CPh_3_) such as BIT^OMe,StBu^ (**2**) had been employed by Nicholas and co-workers in an attempt to inhibit dinuclear Cu complex formation to potentially kinetically stabilize mono-Cu-O_2_ species. However, reactions of the Cu^I^ derivatives afford mainly dinuclear Cu alkoxo and hydroxo complexes and the oxygenation reactivity of the Cu^I^ complexes depends markedly on the carbinol and thioether susbtituents [10]. 

The oxygenation of Cu^I^ complex with ligand BIT^OH,SMe ^affords a dinuclear Cu^II^ compound featuring bridging BIT-alkoxo ligands and terminal –SOMe_2_. The incorporation of methyl in BIT^OMe,SR’ ^ligands prevents the oxidation of the sulfur atom. The different R’ substituent affects the reactivity of the Cu^I^ towards oxygenation and thus sterically hindered ligand BIT^OMe,StBu^ (**2**) affords a slower oxygenation [10]. We choose ligand **2** as a candidate for the preparation of different complexes of Au, Ag and Cu to be applied in homogeneous oxidation reactions as well as to study their biological activity.

Ligand **2** was prepared as previously reported [10] but we isolated and fully characterized precursor ligand BIT^OH,StBu^ (**1**) and prepared **2** from isolated **1** (see the Experimental section). The addition of ligand **2** to solutions of different salts of group 11 metals (see Experimental for details) affords compounds [Au{BIT^OMe,StBu^}Cl][AuCl_4_]_2_ (**3**), [Au{BIT^OMe,StBu^}Cl] (**4**), [Ag{BIT^OMe,StBu^}X] (X = OSO_2_CF_3_^-^ **5**, PF_6_^-^ **6**) and [Cu{BIT^OMe,StBu^}Cl_2_] (**7**) (Scheme 1). Depending on the metals, **2** acts as a pincer ligand (see Scheme 1). All complexes were isolated in moderate yields as air-stable solids. The stoichiometry of the compounds is proposed on the basis of their spectroscopic characterization, microanalysis, mass spectrometry and conductivity studies (see experimental). The geometries for the metal in these derivatives (square-planar for Au^III^, linear for Au^I^ and Ag^I^ and, based on the X-ray structure of related mononuclear [(BITO^Me,SMe^)Cu^II^(DMF)_2_]^2+^[10] square-pyramidal geometry for the Cu(II)) are the most plausible ones considering all these data. It is clear that the two silver compounds **5** with the triflate ligand and **6** with the PF_6_^-^ anion are different. Compound **5** is a neutral compound with the triflate coordinated as a covalent (instead of ionic) group [12], as confirmed by its IR and conductivity measurements (see Experimental). However, polynuclear structures cannot be excluded for the silver derivatives. The monomeric nature of the Cu^II^ derivative [Cu{BIT^OMe,StBu^}Cl_2_] (**7**) was confirmed by EPR studies of solutions of **7** in CH_3_CN at 77 K. The X-band spectrum shows signals that can be interpreted as being due to a paramagnetic entity (*S* ) 1/2) with nearly axial symmetry, whose gyromagnetic tensor was determined by g || = 2.35 and g ⊥ = 2.073, and is typical for monomeric or isolated Cu^II^ complexes [13]. Unfortunately we could not get crystals of enough quality for single crystal X-ray diffraction studies of **4-7**. 

**Figure 1:**
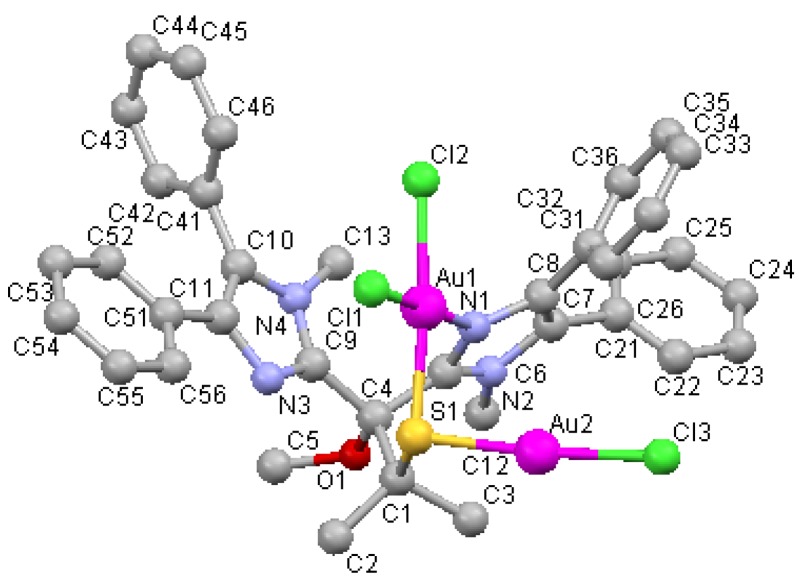
Molecular drawing of neutral dinuclear mixed Au^III^-Au^I^ species [Au_2_{BIT^OMe,S^}Cl_3_] **8** (obtained while crystallizing compound[Au{BIT^OMe,StBu^}Cl][AuCl_4_]_2_ **3**) with the atomic numbering scheme.

We obtained high quality red crystals from CH_2_Cl_2_/Et_2_O solutions of the Au(III) compound [Au{BIT^OMe,StBu^}Cl][AuCl_4_]_2_ (**3**). The structure corresponds to a new neutral dinuclear Au(III)-Au(I) compound (**8** in Scheme 1). The structure is shown in Figure 1, while selected bond and angles are presented in Table 1. The tripodal ligand is coordinated to both a Au^III^ center and a Au^I ^center via the sulfur atom which has lost the *tert*-Bu group becoming a thiolato functional group instead of the original thioether. The Au^III^ ion is four-coordinated in a square-planar geometry as expected with angles close to 90. The Au^I^ is two-coordinated, but the coordination geometry around the gold atom is slightly distorted from linearity, with a S(1)-Au(2)-Cl(3) angle of 174.81(8). The Au^III^-Cl bond lengths are 2.3066(18) and 2.31119(19) Å, similar to those found for the only two crystal structures of mixed Au(III)-Au(I) species with a S atom as a bridge and containing chlorides, [Na(15-crown-5)]_4_[AuCl_2_]_2_[Au_4_Cl_6_S_2_] (2.34(1)-2.31(1)) and [Na(15-crown-5)] [Au_4_Cl_6_S_2_] (2.332(3)-2.328(3)) [14]. The Au^III^-S bond length (2.327(2) Å) compares well with the value found for one of the few Au(III) thiolate complexes described [Au_2_Cl_4_(μ-SPh)_2_] (2.332(5)-2.339(5) Å) [15] and is shorter than those found for mixed Au^III^-Au^I^ thiolate complexes with ligands coordinated to the Au^III^ other than chlorides (e.g., [(C_6_F_5_)_3_Au(μ_2_-2-SC_6_H_4_NH_2_)(AudppmAu)(μ_2_-2-SC_6_H_4_NH_2_)Au(C_6_F_5_)] (2.372(3)-2.375(5) Å) [16], and 2.380(2) [(C_6_F_5_)_3_Au(μ_2_-SC_6_F_5_)(AudppfAu)( μ_2_-SC_6_F_5_)Au(C_6_F_5_)_3_]) [17]. The Au^I^-S and Au^I^-Cl distances of 2.248(2) Å and 2.258(2) Å are very close to those found in the above mentioned crystal structures of [Na(15-crown-5)]_4_[AuCl_2_]_2_[Au_4_Cl_6_S_2_] (2.24(2) and 2.24(1) Å) and [Na(15-crown-5)] [Au_4_Cl_6_S_2_] (2.247(3) and 2.282(2) Å) [14].

**Table 1:** Selected bond distances (Å) and angles (°) in species **8**.

Au1 N1 2.038(5) Å	N1 Au1 Cl1 177.4(2)°
Au1 Cl1 2.3066(18) Å	Cl2 Au1 S1 174.35(8)°
Au1 Cl2 2.3119(19) Å	N1 Au1 Cl2 91.01(17)°
Au1 S1 2.327(2) Å	Cl1 Au1 Cl2 90.66(7)°
Au2 S1 2.248(2) Å	N1 Au1 S1 89.42(17)
Au2 Cl3 2.258(2) Å	Cl1 Au1 S1 89.11(7)°
Au1 Au2 3.383 Å	S1 Au2 Cl3 174.81(8)°
	Au2 S1 Au1 95.37(6)°

The Au^I^-Au^III^ distance is 3.383 Å and it is comparable to the shortest distances obtained in two other bridging thiolate and sulfide mixed Au^I^-A^III^ complexes. Thus the Au^I^-Au^III^ distances obtained for tetranuclear [(C_6_F_5_)_3_Au(μ_2_-SC_6_F_5_)(AudppfAu)(μ_2_-SC_6_F_5_)Au(C_6_F_5_)_3_] are 3.2812(7) and 3.3822(7) Å [3.2923(7) and 3.4052(7) Å] [17] and, in the case of [{S(Au_2_dppf)}_2_{Au(C_6_F_5_)_2_}][CF_3_SO_3_], 3.2923(7) and 3.4052(7) Å [18]. The above mentioned distances (including that in **8**) are shorter than those reported in other sulfide, thiolate, or selenide mixed derivatives [3.404(1)-4.011(1) Å] [17-20]. This distance may be indicative of the presence of a weak interaction between the Au^I^ and Au^III^ centers [17] and is similar to that for Au^I^-Au^I^ contacts in many molecules. 

Although the loss of the *tert*-Bu group in the [Cu(BIT^OMe,StBu^)] complexes was not described, Nicholas *et al*. [10] reported the loss of the trityl group (CPh_3_) from [Cu(BIT^OMe,STr^)] derivatives in reactions of ligand ((BIT^OMe,STr^) and [Cu(CH_3_CN)_4_]PF_6_ with O_2_ or under Ar in the presence of *tert-*BuNC. The compounds obtained were [(BIT^OMe,S^)_2_Cu_2_(μ-OH)_2_](PF_6_)_2_ in the first case or [(BIT^OMe,S^)_2_Cu_2_(^t^BuNC)_2_] and [(BIT^OMe,S^)Cu_2_ (^t^BuNC)_2_]PF_6_ in the second [10]. The authors reported that the loss of trityl group occured before oxygenation. In our case this cleavage occurs with concomitant reduction of one counter ion [AuCl_4_]^-^ to AuCl, which then coordinates to the S atom. Some thioether ligands can afford Au(I) derivatives by reduction of HAuCl_4 _ [21] and the reduction of Au^III^ complexes with calix [4] arenes modified by thioether groups on the upper rim to Au^I^ has been reported as well [22]. Carbon-sulfide bond cleavage in spontaneously adsorbed thioether-based monolayers at gold was described and gold-bound thiolate species were detected [23]. The nature of the N_2_S chelating ligand **2** may allow for a coordination of a counter ion Au^III^Cl_4_^-^ and reduction to Au^I^Cl with a concomitant C-S bond cleavage and loss of *tert*-butyl alcohol. 

### 2.2. Catalysis

Oxidation is a very important method for the synthesis of chemical intermediates in the manufacture of high-tonnage commodities, high-value fine chemicals, agrochemicals and pharmaceuticals. Oxidation of olefins provides a way to turn mineral oil into chemicals of commercial value. Gold has been reported to be a catalyst in homogeneous and heterogeneous [24] oxidation reactions. The number of reports on truly homogeneous catalytic oxidation reactions with gold compounds is more limited. AuCl in combination with nitrogen or phosphorous containing ligands has been used as a catalyst in the aerobic oxidation of alcohols [25] and the oxidative cleavage of double [26], C-H, [27] and single and triple bonds [27]. Very importantly, Au^III^ cations have been used in the oxidation of methane to methanol (in the presence of strong acids) [28,29] while Au^III^-chlorides and [AuCl_2_(NO_3_)(thioether)] complexes have been employed in sulfoxide [30,31] and thioether oxidations [32,33]. Common complexes such as Na[AuCl_4_] or [AuCl(PPh_3_)] have been used in the oxidation of alkanes (with very poor conversions) [34]. AuCl_3_ and AuCl_2_ with NO ligands have been used in the epoxidation of alkenes [35] and [Au(OAc)_3_] in the aerobic oxidation of dibenzylamine [36]. Very recently, oxidative transformations of Au-C(sp^2^) bonds [37] have been reported. In these processes there was strong evidence for the existence of Au^I^/Au^III^ catalytic cycles. This has opened up a new area of gold catalysis where *in situ*-generated Au-C(sp^2^) bonds can be oxidatively transformed in the presence of external oxidants [37]. 

We wanted to study the catalytic activity of the new Au^III^ and Au^I^ complexes with the same set of ligands BIT^OMe,StBu^ (**2**) [10] and chlorides. We chose the reaction depicted in Table 2: the oxidative cleavage of diphenyl ethylene in the presence of catalyst and *tert*-butyl hydroperoxide to produce benzophenone. 

**Table 2:** Oxidative Cleavage of Diphenyl Ethylene. 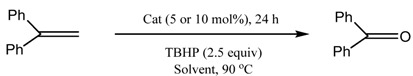

Run	Catalyst	Solvent	Temp. (°C)	Cat. mol %	Conversion % ^a^
1	**3**	Toluene / Acetonitrile	90	5	26
2	**3**	Toluene / Acetonitrile	90	10	31
3	**4**	Toluene	RT	5	0
4	**4**	Toluene	RT	10	0
5	**4**	Toluene	90	5	43
6	**4**	Toluene	90	10	60
7	**5**	Toluene / Acetonitrile	90	5	42
8	**5**	Toluene / Acetonitrile	90	10	48
9	**6**	Toluene / Acetonitrile	90	5	42
10	**6**	Toluene / Acetonitrile	90	10	80
11	**7**	Toluene / Acetone	90	5	0
12	**7**	Toluene / Acetone	90	10	7
13	**9**	Toluene	90	10	10

^a^ By ^1^H-NMR.

AuCl in the presence of neocuproine (5 mol %) in different solvents catalyzed the reaction at 90 °C in 10 hours. The reaction could be run in water and the amount of catalyst and ligand could be decreased to 1 mol %. The replacement for AuCl_3_ salts afforded a very low conversion to benzophenone [26]. In our case, we tested the Au^III^ and Au^I^ compounds **3** and **4** (entries 1-6) in either toluene or mixtures of toluene/acetonitrile for the cationic Au^III^ compound **3**. The compounds were active although the conversions were lower than those described for AuCl and neocuproine. The Au^I^ complex was a better catalyst than the Au^III^ derivative as previously described [26]. The increase of the amount of catalyst improved the conversion but did not double it. Our reaction time was higher (24 h instead of 10 h) and the reactions were run at 90 °C. We also tested the silver derivatives **5** and **6** and found a similar activity to the Au^I^ for the one with covalent triflate (**5**) whereas the cationic silver derivative with PF_6_^-^ anion (**6**) was the most active of all studied with a conversion of 80% (10 mol % cat). Homogeneous oxidation of organic products with silver derivatives is limited to a couple of not very detailed patents with inorganic silver salts (like AgNO_3_) [38,39]. To our knowledge, these are the first examples of well-defined organic soluble silver catalysts in oxidation reactions. In contrast, neither the Cu^I^ or Cu^II^ derivatives showed activity in this oxidative cleavage with TBHP. Cu^I^ complexes of **2** had been described as having a lower reactivity towards oxygen in comparison to other BIT ligands [11].

### 2.3. Biological Activity

#### 2.3.1. Antimicrobial Activity

The antimicrobial activity of the new compounds **3**-**5**, **7** as well as that of compound **9** and the ligand **2** were evaluated against yeast (*Saccharomyces cerevisiae*) Gram-negative (*Escherichia coli and Salmonella tiphymurium*) and Gram-positive (*Bacillus cereus* and *Staphyloccocus aureus*) bacteria (Table 3). 

The ligand {BIT^OMe,StBu^} **2** was totally inactive against *S. cerevisiae* and the representative Gram-positive and Gram-negative bacteria. *S. cerevisiae* showed clearing at the highest concentration tested for gold and silver compounds, but was unresponsive towards the copper compounds; these compounds should be tested against fungi and other microbes to determine general toxicity ranges for eukaryotes. Au^III^ complex **3** showed moderate toxicity toward both Gram-positive bacteria, but had low toxicity towards the Gram-negative bacteria. Though Au^I^ complex **4** showed low toxicity towards most bacteria, *Escherichia coli* was highly vulnerable to this compound, which may be a precursor for an narrow-range antibacterial. Ag^I^ complex **5** was highly toxic to all bacteria tested and is a promising candidate for a broad-range antibacterial. Cu^II^ complex **7** was highly toxic to *Bacillus cereus*, but completely innocuous to other bacteria, implying that a targeted antibacterial can be developed from compound **7**. Cu^I^ complex **9** was not toxic to the bacteria tested in this study.

**Table 3:** Toxicity assesment of compounds **3**-**5**, **7**, **9** and ligand **2** against microbial organisms.^a^

Compound	*Yeast*	*Gram-negative*		*Gram-positive*	
	*S. Cerevisiae (X2180-1A)*	*E. Coli*	*S.* *Tiphymurium*	*B. Cereus*	*S. Aureus*
**2** ^b^	---	---	---	---	---
**3** (Au^III^) ^c^	100	100	100	10	10
**4** (Au^I^) ^c^	100	0.1	100	---	100
**5** (Ag^I^) ^c^	100	10	10	10	10
**7** (Cu^II^) ^c^	---	---	---	10	---
**9** (Cu^I^) ^c^	---	---	---	---	---

^a^ Minimun number of μg required to create a zone of clearing of 0.5 cm diameter on a lawn of fungal or bacterial cells: 1, 10, 100 or --- no toxicity. ^b^ Ligand **2** was dissolved in CH_2_Cl_2_. ^c^ Metallic compounds dissolved in CH_3_CN.

We found that the silver derivative **5** is the most potent against Gram-negative bacteria with the exception of a lower toxicity for the Au^I^ species towards the Gram-negative *Escherichia coli* bacteria. Au^III^ and Ag^I^ derivatives are more toxic towards Gram-positive bacteria than the Au^I^ analogue. This in contrast to results found for Au^I^ complexes containing phosphanes, including our work with Au^I^ water-soluble phosphanes [40]. The fact that gold compounds are more toxic for Gram-positive bacteria than Gram-negative ones or fungi has been noted previously with auranofin [41] and some other Au^I^ phosphane derivatives [40,42] and *N*-heterocyclic carbene-Au^I^ derivatives [43]. Compound **3** is one of the very few examples of Au^III^ derivatives exhibiting antimicrobial properties [44]. Dinuclear Ag^I^-oxygen bonding complexes derived from camphanic acic ligands have displayed a wide spectrum of effective antimicrobial activity [45]. Some N-heterocyclic carbene Ag^I^ complexes inhibit more efficiently the growth of Gram-positive bateria including *B. subtilis* [46,47,48], Gram-negative *E. coli* [47,48,49,50]*, and P. aeruginosa* [47,49,50] and even antibiotic resistant strains of *S. aureus*. [50,51] Binuclear and polymeric Ag^I^ complexes with a tridentate heterocyclic N- and S- ligand 8-(9pyridin-3-yl)methylthio) quinoline were good inhibitors of Gram-positive bacteria and some Gram-negative bacteria (*P. aeruginosa*) but they were very poor against *E. coli* [52]. The behaviour of the silver compound **5 **is similar to that of silver-carbene derivatives, [46,47,48,49,50,51] silver fluorinated tris(pyrazolyl)-borate complexes [53] and AgNO_3 _and silver(I) sulfadiazine [52] inhibiting grow of both Gram-negative and Gram-positive bacteria. However in the case of the tris(pyrazolyl)borate complexes it was demonstrated that the effect on Gram-positive species was due to the ligand whereas the activity for Gram-negative species was truly due to the silver ion [53]. In our case it seems that the activity displayed is due to the presence of the metal. Thus **5** has potential applications in the broad spectrum of antimicrobial field.

#### 2.3.2. Cytotoxicity

The cytotoxic properties of the Au^III^, Au^I^, Ag^I^, Cu^II^ and Cu^I^ compounds were analyzed *in vitro* as previously described utilizing human HeLa cervical carcinoma cell line that expresses the Green Fluorescence Protein (GFP) in the nucleus [54,55]. Before use, all test compounds were dissolved in DMSO and used at a final solvent concentration of 0.1% that has no discernible effect on cell viability. The well known anticancer cytotoxic agent, cisplatin, was used as a positive control for cell death induction. It is apparent that the complexes displayed low to very low cytotoxicity towards HeLa cells. Gold compounds **3**, **4** were 1/10 as effective as cisplatin in causing death of HeLa cells, whereas Ag^I^ (**5**) was 1/5 as effective. The cytotoxicity of **6** could not be evaluated due to the formation of crystals in the culture media. Due to the low solubility of the copper compounds **7**, **9** in DMSO-H_2_O, their toxicity towards HeLa cells was very low (less than 10%) and could not be fully evaluated. If the values obtained are an indication of their cytotoxic potential at higher concentrations they would still fall far below the toxicity values of the other compounds and cisplatin.

**Figure 2:**
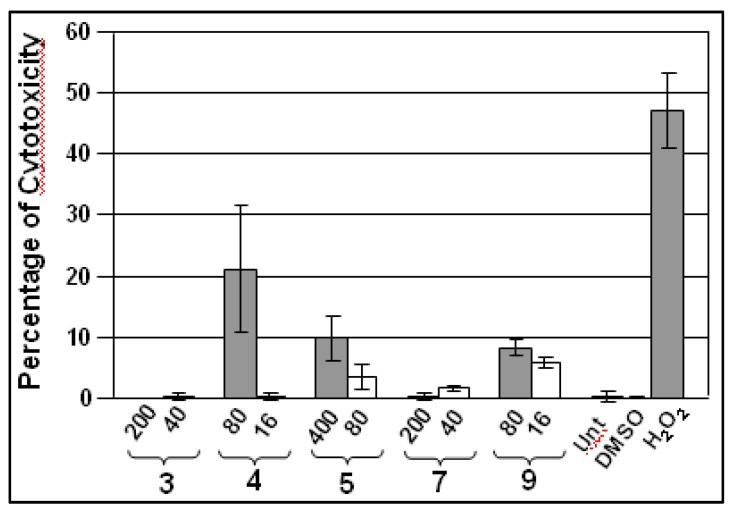
*In vitro* cytotoxicity of compounds **3**-**5**, **7** and **9** against normal cells (conc. μM). Cytotoxicity was monitored by using live-cell imaging of a normal primary culture of adherent cells obtained from C57B6 mouse muscle explants after 22 hrs of incubation with the compounds. Each bar represents average value of three measurements and error bars represent standard deviations. Results for compound **3** at 200 µM are not shown since there was no cytotoxicity observed. Three controls were included; untreated cells (Unt), treated with 0.5% v/v diluent of the chemical compounds (DMSO) and treated with 1 mM H_2_O_2_.

Moreover, we studied the cytotoxicity of compounds **3**-**5**,**7** and **9** against normal primary cells (Figure 2). Cytotoxicity was monitored by using live-cell imaging of a normal primary culture of adherent cells obtained from C57B6 mouse muscle explants after 22 hrs of incubation with the compounds. The cytotoxicity of these compounds against the normal cells was very low although there were some differences since complexes of Au^I^ **4** and Cu^I^ (**9**) were slightly more toxic than the Au^III^ (**3**), Ag^I^ (**5**) and Cu^II^ (**7**) derivatives. 

This lack of toxicity in cancerous and normal mammalian cell lines makes them promising candidates as potential antimicrobial agents (low toxicity in human tissues) and as safer and greener catalysts. 

**Table 4:** IC_50_ values for HeLa-GPF cells of compounds **3**-**7** and ligand **2** [10] and complex **9** [10], compared to cisplatin.

	IC_50_(μM) ^a^							
Cell line	**Cisplatin**	**2 ^b^**	**3**	**4**	**5**	**6 ^b^**	**7** ^c^	**9 ** ^c^
HeLa-GPF	14.9	----	155	155	73.6	---	---	---

^a^ IC_50_ is defined as the concentration of drug required to disrupt the plasma membrane of 50% of cell population, compared to untreated cells, after 22 hours of incubation. Cells with compromised plasma membrane were monitored using propidium iodide (PI) and flow cytometry. Cisplatin was used as reference compound. ^b^ Compound **6** was found to be soluble in DMSO but when 1 µL was added to culture media with cells the compound came out of solution forming crystals. Since it was difficult to evaluate how much of the compound was actually in solution, an accurate IC_50_ value could not be determined. In the case of compound **2**, it was possible to examine its cytotoxicity against HeLa-GFP cells at 250 µM, but its cytotoxicity was very poor (5.4 ± 2.1%). ^c^ Due to the low solubility of the copper compounds in the DMSO-H_2_O mixture, the maximum concentration possible tested for **7 **and **9** was 50 and 25µM, and their percent of cytotoxicity was 7.4 and 9, respectively.

## 3. Experimental

### 3.1. General

Solvents were purified by use of a PureSolv purification unit from Innovative Technology, Inc. (Newburyport, MA, USA); all other chemicals were used as received. Elemental analyses were carried out by Atlantic Microlab, Inc. (Norcross, GA, USA). Infrared spectra (4000-400 cm^−1^) were recorded on a Nicolet 380 FT-IR infrared spectrophotometer on KBr pellets. The ^1^H-, and ^13^C{^1^H}-NMR spectra were recorded in CDCl_3_ solutions at 25 °C on a Bruker 400 and spectrometer (δ, ppm; J, Hz); ^1^H and ^13^C{^1^H} were referenced using the solvent signal as internal standard. EPR studies were conducted on a Bruker E500 EPR spectrometer. The mass spectra (ESI) were recorded from CH_2_Cl_2_ or CH_3_CN solutions by the mass spectrometry facility of the University of California Riverside (Riverside, CA, USA). X-ray data were collected with a Nonius Kappa CCD (Hunter College, The City University of New York, New York, NY, USA). Conductivity was measured in an Oakton pH/conductivity meter. Ligand **2** was prepared as previously reported [10], but we isolated and characterized precursor ligand BIT^OH,StBu^ (**1**) and prepared **2** from isolated **1**. All other chemicals and solvents were purchased from Sigma-Aldrich and Strem Chemicals. 

*1-Methoxy-2-methyl-1,1-bis(N-methyl-4,5-diphenyl-2-imidazolyl)2-tert-butylthiopropanol* (**1, BIT^OH,StBu^**). To a solution of N-methyl-4,5-diphenylimidazole (1.40 g, 6.0 mmol) in 30 mL of dry THF under N_2_ cooled to −78 °C was added *n*-BuLi dropwise (2.5 M, 2.40 mL, 6.0 mmol). The mixture was stirred at −78 °C for 3 h to give a red-brown solution. After the addition of ethyl-2-methyl-*tert-*butylthio-propanonate (0.41 g, 3.0 mmol), the mixture was allow to warm to room temperature overnight to give a yellow solution. The reaction was quenched with 80 mL of water and extracted with diethyl ether (20 mL) three times. The organic phase was dried over MgSO_4_, filtered through Celite, and vacuum dried to give **1** as a pure white compound. Yield: 1.40 g, 75%. Anal. Calc. for C_40_H_42_N_4_OS (626.92): C, 75.71; H, 6.88; N, 8.80; S, 4.78; found: C, 75.74; H, 6.68; N, 8.76; S, 4.54. MS (ESI+) [*m/z*]: 627.3 [M+1]^+^. IR (cm^−1^): ν (C=N, C_3_N_2_) = 2958, ν (C-N, C_3_N_2_) = 1501, ν (C-OCH_3_) = 1074, ν (S-C (CH_3_)_2_) = 970. ^1^H-NMR: δ = 7.18−7.68 (m, 20H, H_p_+H_o_+H_m_, C_6_H_5_ Bz); 5.70 (s, 1H, OH); 3.08 (s, 6H, N-CH_3_); 2.28 (s, 6H, SC (CH_3_)_2)_; 1.49 (s, 9H, C (CH_3_)_3_). ^13^C{^1^H}-NMR: δ = 15.24 [C(CH_3_)_3_], 27.59 (N-CH_3_), 32.05 [SC(CH_3_)_2_], 33.46 [C(CH_3_)_3_], 47.28 [SC(CH_3_)_2_], 59.73 (O-CH_3_), 126.17 (C_3_, C_3_N_2_), 126.50 (C_2_, C_3_N_2_), 128.04 (C_o_, Ph), 128.08 (C_p_, Ph) 131.07 (C_m_, Ph), 134.67 (C_1_, Ph), 146.25 (C_1_, C_3_N_2_).

*1-Methoxy-2-methyl-1,1-bis(N-methyl-4,5-diphenyl-2-imidazolyl)2-tert-butylthiopropane* (**2, BIT^OH,StBu^**) [10] can be prepared from isolated **1** in 80% yield as follows: to a suspension of sodium hydride (0.048 g, 1.92 mmol) in 10 mL of dry THF under N_2_ was added the solution of **1** (1.003 g, 1.6 mmol) in 10 mL of dry THF, and the resulting mixture was stirred for 1 h at room temperature. Methyl iodide (0.12 mL, 1.92 mmol) was added dropwise, and then the reaction mixture was stirred at reflux under N_2_ for 22 h. The reaction mixture was quenched with 80 mL of water and extracted with dichloromethane (20 mL) three times. The organic phase was dried over MgSO_4_, filtered through celite, and vacuum dried to give **2** as a yellow powder. Yield: 0.870 g, 80%.

*[Au{BIT^OMe,StBu^}Cl][AuCl_4_]_2_* (**3**): To a solution of K[AuCl_4_] (0.166 g, 0.44 mmol) in acetonitrile (2 mL) a solution of BIT^OMe,StBu ^(**2**, 0.140 g, 0.22 mmol) in CH_2_Cl_2_ (20 mL) was added dropwise at 0 °C. The resulting solution was allowed to stir for over 40 min at RT and the solvent was removed under vacuum to ca. 1 mL. Addition of Et_2_O/n-hexane (1:1,15 mL) and cooling to −5 °C afforded **3** as a pale orange solid that was filtered and dried under vacuum. Yield: 0.213 g, 62%. Anal. Calc. for C_41_H_44_N_4_OSAu_3_Cl_9_ (1550.85): C, 31.75; H, 3.61; N, 2.07; found: C, 32.04; H, 3.17; N, 3.69; MS (ESI+) [*m/z*]: 641.3 [Ligand -H]^+^. IR (cm^−1^): ν(C = N, C_3_N_2_) = 2958, ν(C-N, C_3_N_2_) = 1505, ν(C-OCH_3_) = 1090, ν[S-C(CH_3_)_2_] = 973. ^1^H-NMR: 7.18-7.54 (m, 20H, H_p_+H_o_+H_m_, C_6_H_5_ Ph); 3.78 (s, 3H, O-CH_3_) 3.47(s, 3H, N-CH_3_); 2.76 (s, 3H, N-CH_3_); 1.65 [s, 6H, SC (CH_3_)_2_]; 1.28 [s, 9H, C (CH_3_)_3_]. ^13^C{^1^H}- NMR: δ = 24.37 [C(CH_3_)_3_], 28.12 (N-CH_3_), 29.53 [SC(CH_3_)_2_], 33.51 [C(CH_3_)_3_], 36.44 [SC(CH_3_)_2_], 45.80 (COCH_3_), 93.27 (COCH_3_), 126.70 (C_3_, C_3_N_2_), 127.99 (C_2_, C_3_N_2_), 128.94 (C_p_, Ph), 129.45 (d, C_m_, Ph) 129.82 (C_o_, Ph), 130.53 (C- C_3_N_2_, Ph), 173.73 (C_1_, C_3_N_2_). Λ_(CH3CN)_ = 321 Ω^−1^cm^2^mol^−1^, 3 ions (1+/2-). 

*[Au{BIT^OMe,StBu^}Cl]* (**4**): To a solution of [AuCl(tht)] (0.032 g, 0.10 mmol) in CH_2_Cl_2_ (10 mL) a solution of BIT^OMe,StBu ^(**2**, 0.064 g, 0.10 mmol) in CH_2_Cl_2_ (10 mL) was added dropwise at 0 °C. The resulting solution was allowed to stir for over 40 min at RT and the solvent was removed under vacuum to ca. 1 mL. Addition of Et_2_O/n-hexane (1:1, 15 mL) and cooling to −5 °C afforded **4** as a white solid that was filtered and dried under vacuum. Yield: 0.045 g, 69%. Anal. Calc. for C_41_H_44_N_4_OSAuCl (873.30): C, 56.39; H, 5.08; N, 6.42; found: C, 56.78; H, 5.16; N, 6.50; MS (ESI+) [*m*/*z*]: 873 [M + 1]. IR (cm^−1^): ν(C=N, C_3_N_2_) = 2954, ν(C-N, C_3_N_2_) = 1501, ν(C-OCH_3_) = 1087, ν [S-C(CH_3_)_2_] = 960. ^1^H-NMR: δ =7.18-7.54 (m, 20H); 3.75 (s, 3H); 3.75 (s, 3H); 3.41 (s, 6H); 1.65 (s, 6H) 1.34 (s. 9H). ^13^C{^1^H}-NMR: δ = 23.69 [C(CH_3_)_3_], 27.62 (N-CH_3_), 28.02 [SC(CH_3_)_2_], 33.46 [C(CH_3_)_3_], 47.41 [SC(CH_3_)_2_], 55.05 (COCH_3_), 88.37 (COCH_3_), 125.73 (C_3_, C_3_N_2_), 126.56 (C_2_, C_3_N_2_), 127.94 (C_o_, Ph), 128.96 (C_m_, Ph), 130.53 (C_p_, Ph), 138.27 (C_1_, C_3_N_2_). Λ_(CH3CN)_ = 17 Ω^−1^cm^2^mol^−1^, neutral.

*[Ag{BIT^OMe,StBu^}X]* (X = OSO_2_CF_3_ **^-^ 5, **PF_6_^-^ **6**): To a solution of AgOSO_2_CF_3 _(0.026 g, 0.1 mmol, **5**) or AgPF_6_ (0.075 g, 0.3 mmol, **6**) in Et_2_O or CH_3_CN (5 mL) a solution of BIT^OMe,StBu^ (**2**) (0.064 g, 0.10 mmol, **5** or 0.192 g, 0.3 mmol, **6**) in CH_2_Cl_2_ (10 mL) was added dropwise at 0 °C. The resulting mixtures were allowed to stir for over 40 min at 0 °C protected from light exposure. Subsequent removal of the solvent under vacuum to ca. 1 mL, addition of Et_2_O/n-hexane (1:1, 15 mL) and cooling to −5 °C afforded **5** and **6** as a white solids that were filtered and dried under vacuum. Compound **5**: yield: 0.050 g, 56%. Anal. Calc. for C_42_H_44_N_4_OS_2_AgF_3_ (897.81): C, 56.57; H, 4.29; N, 6.28; S, 7.19 found: C, 56.13; H, 4.67; N, 5.93; S, 7.29. MS (ESI+) [*m*/*z*]: 748.22 [M]^+^. IR (cm^−1^): ν(C = N, C_3_N_2_) = 2953, ν(C-N, C_3_N_2_) = 1505, ν(SO) = 1248 (br, s), 1157 (s), 1023 (m), ν(S-CF_3_) = 640 (signals of C-OCH_3_ and S-C(CH_3_)_2_ have been overlapped by the signals of covalent OSO_2_CF_3_). ^1^H-NMR: δ = 7.23−7.74 (m, 20H, H_p_+H_o_+H_m_, C_6_H_5_ Ph); 4.24 (t, 6H, N-CH_3_); 1.36 [s, 6H, SC(CH_3_)_2_]; 0.99 [s, 9H, C(CH_3_)_3_]. ^13^C{^1^H}-NMR: δ =14.06 [C(CH_3_)_3_], 25.36 (N-CH_3_), 28.18 [SC(CH_3_)_2_], 33.48 [C(CH_3_)_3_], 35.79 [SC(CH_3_)_2_], 57.05 (COCH_3_), 61.14 (COCH_3_), 126.59 (C_3_, C_3_N_2_), 127.92 (C_2_, C_3_N_2_), 128.93 (C_o_, Ph), 129.76 (C_m_, Ph), 131.12 (C_p_, Ph), 132.81(C_1_, C_3_N_2_). Λ_(CH3CN)_ = 93 Ω^−1^cm^2^mol^−1^, neutral. Compound **6**: yield: 0.153 g, 60%. Anal. Calc. for C_43_H_47_N_5_SOAgPF_6_ (934.77): C, 55.25; H, 5.07; N, 7.49; found: C, 55.01; H, 4.90; N, 7.04. MS(ESI+) [*m*/*z*]: 747.22 [M -1]^+^. IR (cm^-1^): ν(C=N, C_3_N_2_) =2958, ν(C-N, C_3_N_2_) = 1501, ν(C-OCH_3_) = 1074, ν(S-C(CH_3_)_2_) = 979. ^1^H-NMR: δ = 7.58-7.27 (m, 20H, H_p_+H_o_+H_m_, C_6_H_5_ Ph); 3.95 (s, 3H, N-CH_3_); 3.05 (br, 3H, O-CH_3_); 2.62 (s, 3H, N-CH_3_); 1.64 [s, 6H, SC (CH_3_)_2_]. ^13^C{^1^H}-NMR: δ =14.07 [C(CH_3_)_3_], 22.62 (N-CH_3_), 31.21 [SC(CH_3_)_2_], 33.51 [C(CH_3_)_3_], 35.67 [SC(CH_3_)_2_], 51.69 (COCH_3_), 82.58 (COCH_3_), 126.41 (C_3_, C_3_N_2_), 128.09 (C_2_, C_3_N_2_), 128.70 (C_o_, Ph), 129.40 (C_m_, Ph), 131.39 (C_p_, Ph), 144.16 (C_1_, C_3_N_2_). ^31^P{^1^H}-NMR (CDCl_3_): -144.32 (sept). Λ_(CH3CN)_ = 220 Ω^−1^cm^2^mol^−1^, 2 ions (1+/1-).

*[Cu{BIT^OMe,StBu^}Cl_2_]* (**7**): To a solution of CuCl_2_ (0.0134 g, 0.10 mmol) in CH_3_CN (2 mL) a solution of BIT^OMe,StBu^ (**2**) (0.064 g, 0.10 mmol) in CH_2_Cl_2_ (10 mL) was added dropwise. The resulting yellow-green solution was allowed to stir for over 1.5 h at RT and the solvent was removed under vacuum to ca. 1 mL. Addition of Et_2_O/n-hexane (1:1, 15 mL) afforded **7** as a green solid that was filtered and dried under vacuum. Yield: 0.049 g, 63%. Anal. Calc. for C_41_H_44_N_4_OSCuCl_2_.2H_2_O (811.38): C, 60.69; H, 5.96; N, 6.90; found: C, 60.67; H, 5.46; N, 6.92; MS(ESI+) [*m*/*z*]: 738 [M-Cl - 1]+. IR (cm^−1^): ν(C=N, C_3_N_2_) =2958, ν(C-N, C_3_N_2_) = 1501, ν(C-OCH_3_) = 1087, ν[S-C(CH_3_)_2_] = 976. Λ_(CH3CN)_ = 71.5 Ω^−1^cm^2^mol^−1^, neutral.

### 3.2. Catalytic Experiments

Typical procedure: To a solution of metallic complexes **3**-**7**, **9** (amounts as specified in Table 2) in toluene (5 mL) or in toluene (3 mL) – acetonitrile (2 mL) or in toluene (3 mL) - acetone (2 m), a solution of biphenyl ethylene (88 μL, 0.5 mmol) and TBHP (0.171 mL, 70% solution in *t*-BuOH, 1.2 mmol) were added at room temperature. The reaction mixture was stirred at 90 °C for 24 h. The conversion of benzophenone was determined by ^1^H-NMR after work-up of the mixture and isolation of the crude product.

### 3.3. Single-Crystal X-ray Diffraction Studies

Crystals of **8** (red prisms) where obtained when a solution of **3** in CH_2_Cl_2_ was crystallized by slow diffusion of Et_2_O at 0 °C. The intensity data for **8 **were measured with a Bruker–Nonius Kappa CCD diffractometer (graphite monochromated Mo-*K*α radiation, *λ* = 0.71073 Å, φ-ω scans) at 100(2) K (Table 1). The data were not corrected for absorption. Details of the solution and refinements for this compound are presented below: the crystal of **8**, with approximate dimensions 0.36_0.06_0.02 mm, was orthorhombic with space group *P_212121_*. The final unit-cell constants of **8 **were *a* = 11.032(2) Å, *b* = 18.178(4) Å, *c* = 18.202(4) Å, *V* = 3650.2(13) Å3, *Z* = 4, *ρ* = 1.973 gcm−1, *μ* = 8.342 mm−1. The structure of **8 **was solved with SHELXS-97 and refined by full-matrix least-squares on *F*2 with SHELXL-97. The hydrogen atoms were included in the structure-factor calculations, but their parameters were not refined. The final discrepancy indices for the 8373 reflections (*θ* _ 27.52°) were *R* = 0.0464 (calculated on *F*) and *Rw* = 0.0929 (calculated on *F*2) with 438 parameters varied. Tables of thermal parameters and observed and calculated structure factors (crystal structure of compound **8**) have been deposited at the Cambridge Crystallographic Data Center. Any request for this material should quote a full literature citation and the reference number CCDC 784217 and may be obtained from The Director, CCDC, 12 Union Road, Cambridge CB2 1EZ, UK (Fax: +441233336033; Email: deposit@ccdc.cam.ac.uk or www: http://ccdc.cam.ac.uk ). 

### 3.4. EPR studies

The electron paramagnetic resonance (EPR) spectra was measured on a Bruker E500 EPR spectrometer operating at X-band. In the experiment, an ER4122SHQE cavity was used. A solution of **7** (1 mM) in CH_3_CN at 77 K was introduced in a standard EPR quartz tube (707-SQ from Wilmad) and was measured at 77 K using an immersion quartz Dewar. The microwave frequency was determined. The X-band conditions were as follows: 3320 G, 1.0 mW microwave power, 9.51 GHz microwave frecuency, 23dB attenuation, 77 K, 4.0 G modulation amplitude, 100 kHz modulation frequency, 60 dB gain; 163 ms conversion time, 167 s sweep time. EPR data acquisition and manipulation were performed using *XeprView* software (Bruker). CuSO_4_ in 50% ethylene glycol was used as a standard for spin quantification by double integration of EPR signal intensities. The signal-to-noise ratio in EPR spectra was improved by signal averaging when necessary.

### 3.5. Antimicrobial Assays

The gold compounds were tested for microbial toxicity in a Kirby-Bauer disk diffusion assay. Metal compounds **3**-**7** and **9** were dissolved in acetonitrile and ligand **2** (insoluble in CH_3_CN) in CH_2_Cl_2_ to a concentration of 10 mg/mL and serially diluted in methanol by factors of 10 to create solutions ranging down to 0.1 mg/mL. A solution (10 μL) was aliquoted onto a paper filter disk (5 mm diameter × 1 mm thickness) that were then vacuum dried and stored at −20 °C prior to the experiment. For the assay, a cell suspension (100 μL) containing 3 × 10^7^ cells/mL were spread uniformly on a Mueller-Hinton (MH for bacteria) or Yeast Extract (YPD for fungi) agar plate (100 mm × 15 mm). After spreading four paper disks impregnated with either 100, 10, 1, or 0.1 μg of a compound were placed on the agar surface with a solvent control in the center of the plate. The plates were incubated at either 30 °C (fungi) or 37 °C (bacteria) for 48 h and resulting zones of growth suppression were measured.

### 3.6. Cytotoxicity Assays

Adherent HeLa-GFP human cervical carcinoma cells (Kanda *et al.*) [56] were seeded, in a 24 well plate format, 100,000 cells/well using DMEM media (1 mL, HyClone, Logan, UT) supplemented with antibiotics and 10% heat-inactivated newborn calf serum (HyClone). After overnight incubation, to allow cell attachment, they were exposed for 22 h to several concentrations of chemical compounds. Floating cells were collected in a ice-cold tube and placed on ice, while attached cells were treated for 15 min with 0.25% of trypsin solution (Invitrogen, Carlsbad, CA, USA), diluted in serum free DMEM, and incubated at 37 °C. Cells from each individual well, included both those harvested by trypsinization and those floating, were centrifuged at 1,400 rpm for 5 min at 4 °C The media was then removed and cells resuspended in staining solution (500 µL) containing propidium iodide (2 µg/mL) dissolved in FACS buffer (PBS, 0.5 mM EDTA, 2% heat inactivated fetal bovine serum, and 0.1% sodium azide), incubated in the dark at room temperature for 15 min and analyzed by flow cytometry, using a Cytomic FC 500 cytometer (Beckman-Coulter, Miami, FL, USA). The data were analyzed using CXP software (Beckman-Coulter).

### 3.7. Cytotoxicity against normal primary culture cells

Mouse muscle cultures were initiated using tissue explants [57] from healthy 8 to 10 weeks old C57B6 mice utilizing DMEM media supplemented with 10% fetal bovine serum and antibiotics (also referred as complete media). After approximately two weeks of culture, when abundant growth of cells had appeared from the explants, extensive washes with fresh media were performed to eliminate floating cells to selectively keep the adherent cells. The morphology of the adherent cells was mostly of fibroblastic nature (Figure 3). Adherent cells were collected by trypsinization, counted and seeded at a density of 10,000 cells per well in 200 µL of complete media using a 96 well plate format, incubated overnight to promote cell adherence, and incubated for 22 h in the presence of compounds. A mixture of two fluorescent dyes, Propidium iodide (PI; MP Biomedicals, Solon, OH, USA) and Hoechst 33342 (Invitrogen, Eugene, OR, USA) at a final concentration of 1 µg/mL each was added to treated cells 1 h prior to image capture from each individual well utilizing a BD Pathway 855 Bioimager system (BD Biosciences Rockville, MD, USA) [58]. While Hoechst dye has the ability to easily cross cell membranes of healthy and dead cells and stains nuclear DNA, PI is only able to stain dead or dying cells with compromised plasma membranes. The fluorescence signal emitted from each individual fluorophore was captured in two separate channels, according with the dye emission requirements. To acquire adequate cell numbers of cells for statistical analyses, images from nine contiguous image fields (3X3 montages) were captured per well utilizing a 20x objective. Data analysis determining the percentage of death cells from each individual well was performed by using BD AttoVision™ v1.6.2 software [58]. 

## 4. Conclusions

In conclusion, we have prepared new stable complexes of group 11 metals with the previously described tripodal bis(imidazole) thioether ligand (*N*-methyl-4,5-diphenyl-2-imidazolyl)_2_C(OMe) C(CH_3_)_2_S(*tert*-Bu) ({BIT^OMe,StBu^}, **2**) [10] which offers a pincer N_2_S donor atom set. The gold and, especially silver derivatives are active in the oxidative cleavage of diphenylethylene with TBHP at 90 °C. The use of modified BIT ligands (for which it is known that oxygenation reactivity is much higher for Cu^I^ derivatives) [11] may provide more efficient catalysts for this and other homogeneous oxidation processes. The antimicrobial activity of the ligand **2** and the metal complexes has been evaluated against Gram-positive and Gram-negative bacteria and yeast. The new gold and silver compounds display moderate to high antibacterial activity which is not due to the free ligand. Gold and silver complexes are also potent against fungi. Moreover, silver compound **5** seems to hold potential applications in the broad spectrum of antimicrobial field. The fact that the compounds display very low or no cytotoxicity on HeLa Human cervical carcinoma cells and normal mammalian cells makes them promising candidates as potential antimicrobial agents (low toxicity in human tissues) and as safer and greener catalysts. 
